# P052: Molecular epidemiology of methicillin-resistant Staphylococcus aureus (MRSA) strains at Geneva University Hospitals (HUG) over a 9 year period

**DOI:** 10.1186/2047-2994-2-S1-P52

**Published:** 2013-06-20

**Authors:** C Fankhauser, J Schrenzel, P François, G Renzi, D Pittet, S Harbarth

**Affiliations:** 1 Infection Control Program, Geneva University Hospitals, Geneva, Switzerland; 2 Bacteriology Laboratory, Geneva University Hospitals, Geneva, Switzerland; 3 Genomic Research Laboratory, Geneva University Hospitals, Geneva, Switzerland

## Introduction

Changes within the MRSA population of single hospitals have been observed with certain clones replacing others. Surveillance of the genetic diversity within a hospital provides useful epidemiological data. Staphylococcal chromosomic cassettes (SCCmec) of MRSA isolates are routinely determined at HUG.

## Objectives

To describe secular trends of the predominant MRSA clones retrieved at HUG from 2003-12 using SCCmec genotyping.

## Methods

Since 2005, the SCCmec in MRSA isolates (screening swabs or clinical samples) were routinely assessed by multiplex PCR assay. MLVA was used to evaluate genomic diversity; representative isolates were grouped in MLVA clusters and subjected to MLST. All patients’ 1^st^ positive MRSA isolate/year was analysed. Frequency distributions and hospital-acquired (HA)-MRSA rates were determined.

## Results

HA-MRSA cases/100 admissions increased from 1.36 (2000) to 2.00 (2006) and declined to 0.79 (2012). Overall, 9717 MRSA isolates underwent SCCmec genotyping. SCCmecI, the predominant cassette during the study period, peaked at 88% in 2003 and declined to 64% in 2012. SCCmecIV increased modestly from 10% to 16%, followed by SCCmecII with a strong increasing trend from 2% to 14%. Other types were minor contributors (SCCmecV, 3.4%; others <2%). Patients harboring SCCmecI and SCCmecII were older (median age, 76 and 82 y) compared to those with SCCmecIV (60 y). Strain distribution differed by hospital sectors. While SCCmecI remained the prevalent subtype in acute care (AC) and non-AC settings, the proportion of MRSA containing SCCmecII and SCCmecIV was higher in non-AC settings. SCCmecII increased from 6% to 32% in geriatrics. Genotyping confirmed ST228 South German-SCCmecI as the predominant clone. ST105 (CC5) appears to be the predominant clone of SCCmecII. Individual SCCmec replacement was observed in 123 patients (incl. 37 within SCCmecI-SCCmecII and 69, SCCmecI-SCCmecIV).

## Conclusion

ST 228 SCCmecI is still the predominant clone in all settings, but decaying. The prevalence of SCCmecII is higher in geriatrics than in AC settings. The emerging SCCmecII predominant clone is ST105(CC5).

## Disclosure of interest

None declared.

